# MicroRNA‐21 affects mechanical force–induced midpalatal suture remodelling

**DOI:** 10.1111/cpr.12697

**Published:** 2019-11-12

**Authors:** Mengying Li, Zijie Zhang, Xiuge Gu, Ye Jin, Cheng Feng, Shuangyan Yang, Fulan Wei

**Affiliations:** ^1^ Shandong Provincial Key Laboratory of Oral Tissue Regeneration School of Stomatology Shandong University Jinan China; ^2^ Department of Orthodontics School of Stomatology Shandong University Jinan China; ^3^ Jinan ethnic hospital Jinan China

**Keywords:** bone remodelling, mineralized tissue, osteoblast, osteoclast

## Abstract

**Objectives:**

miR‐21 can promote osteoblast differentiation of periodontal ligament stem cells. However, the effect of miR‐21 on bone remodelling in the midpalatal suture is unclear. This study aimed to elucidate the effects of miR‐21 on the midpalatal suture bone remodelling by expanding the palatal sutures.

**Materials and methods:**

miR‐21 deficient (miR‐21^−/−^) and wild‐type (WT) mice were used to establish animal models by expanding the palatal sutures. Micro‐CT, haematoxylin‐eosin (HE) staining, tartrate‐resistant acid phosphatase (TRAP) staining, fluorescence labelling and immunohistochemistry were used to investigate the function of miR‐21 in midpalatal suture bone remodelling. Besides, bone mesenchymal stem cells (BMSCs) derived from both miR‐21^−/−^ and WT mice were cultured. The MTT, CCK8, EdU analysis, transwell and wound healing test were used to assess the effects of miR‐21 on the characteristics of cells.

**Results:**

The expression of ALP was suppressed in miR‐21^‐/‐^ mice after expansion except 28 days. The expression of Ocn in WT mice was much higher than that of miR‐21^‐/‐^ mice. Besides, with mechanical force, miR‐21 deficiency downregulated the expression of Opg, upregulated the expression of Rankl, and induced more osteoclasts as TRAP staining showed. After injecting agomir‐21  to miR‐21^‐/‐^ mice, the expression of Alp, Ocn and Opg/Rankl were rescued. In vitro, the experiments suggested that miR‐21 deficiency reduced proliferation and migration ability of BMSCs.

**Conclusions:**

The results showed that miR‐21 deficiency reduced the rate of bone formation and prolonged the process of bone formation. miR‐21 regulated the bone resorption and osteoclastogenesis by affecting the cell abilities of proliferation and migration.

## 1 INTRODUCTION

Maxillary transverse deficiency (MTD), a common skeletal deformity of craniofacial region,^1^ might lead to occlusal disharmony and functional problems involving breathing patter anomalies.^2^, ^3^ Rapid maxillary expansion (RME) is an effective procedure in orthodontics to increase upper arch transverse dimensions by opening the midpalatal suture.^4^, ^5^, ^6^

However, extensive relapse after RME has been reported, which indicates the limitation of the long‐term effect of RME.^7^, ^8^ The relapse that does occur is considered a result of the resistance to deformation from circum‐maxillary sutures and surrounding soft tissue matrix, inadequate bone formation, and the resorption of new bone formed in the tension area.^9^, ^10^, ^11^ Therefore, it is of utmost importance to explore an effective method for inhibiting bone resorption and accelerating bone formation. At present, numerous studies on promoting bone remodelling during RME have been focused on growth factors, drugs and physical stimulation.^12^, ^13^, ^14^, ^15^ Whether there are factors regulating RME and mediating palatal bone remodelling at post‐transcriptional level remains unknown.

microRNA (miRNA), a type of small non‐coding RNA, has been reported as a critical post‐transcriptional modulator in bone remodelling.^16^ Furthermore, many studies have revealed that some miRNAs, including miR‐21, can regulate osteogenic differentiation as a response to the mechanical stimuli.^17^ Our previous study found that the expression of miR‐21 in human periodontal ligament stem cells (PDLSC) with mechanical stimuli was significantly different from that in non‐stressed ones.^18^ Based on bioinformatics prediction, the target genes of miR‐21 are significantly enriched in Jak‐STAT signalling pathways and MAPK signalling pathways, which are related with osteogenic differentiation.^18^ In addition, our previous work confirmed that miR‐21 can promote stretch‐induced PDLSC osteogenesis by acting on activin receptor 2B (ACVR 2B) in vitro.^19^ Besides, miR‐21 can also promote the differentiation of osteoclasts induced by the receptor activator of nuclear factor κB ligand (RANKL) *in vitro*.^20^, ^21^ In vivo, Chen and his colleagues reported that miR‐21 plays an important role in alveolar bone remodelling, including osteogenic differentiation of periodontal ligament stem cells and osteoclast differentiation.^22^ Nevertheless, the in vivo function of miR‐21 in the regulation of palatal bone remodelling at the post‐transcriptional level, particularly in response to orthopaedic force, remains elusive.

The present study, therefore, aimed to detect the influence of miR‐21 on palatal bone remodelling in mice by analysing the physical and metabolic changes of midpalatal suture in both normal and mechanical stimuli environments.

## 2 MATERIALS AND METHODS

We have already conformed to the ARRIVE guidelines.

### 2.1 Animals and genotyping

Wild‐type (WT) C57BL/6 mice and miR‐21^−/−^ mice were provided by Animal Experimental Center Shandong University and Jackson Laboratory, respectively. Animal experiments were performed based on the guidelines of the Animal Use and Care Committee of Shandong University. Mice were housed in a well‐ventilated room with 12h/12h light‐dark cycle. Food and water were offered ad libitum.

**Figure 1:**
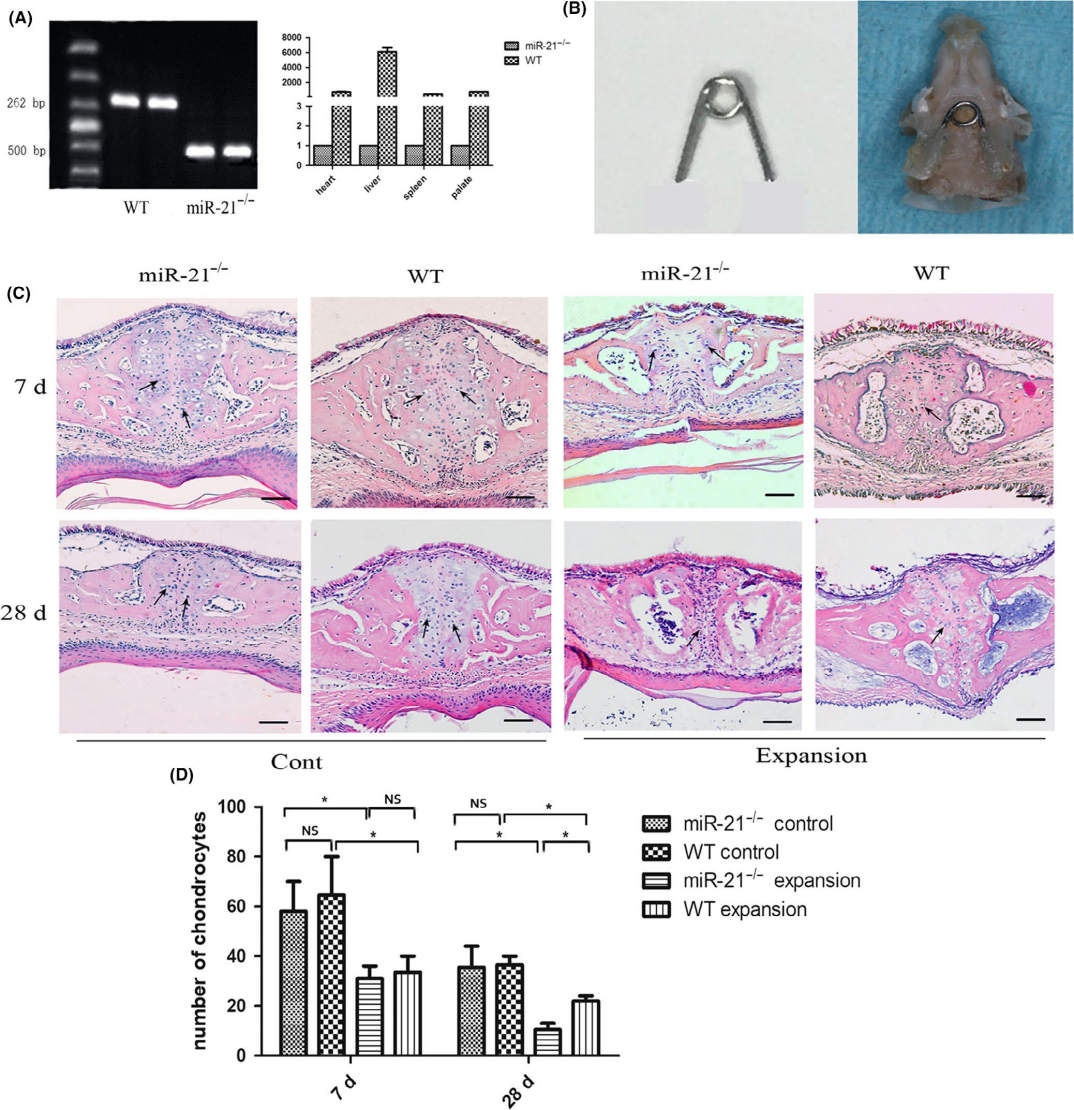
Changes in suture morphology. A, Agarose gel electrophoresis and quantitative real‐time polymerase chain reaction analysis demonstrated miR‐21 was knocked out successfully. B, Occlusal view of mouse maxilla during expansion with the opening loop bonded to molars. C, HE staining in experimental/control group. Mice were subjected to the expansion force for 7 and 28 d. Arrows: chondrocytes. Bars: 50 μm. D, The number of chondrocytes in different groups according to HE staining. miR‐21, microRNA‐21

Standard polymerase chain reaction (PCR) genotyping for WT and miR‐21^−/−^ mice was performed as previously described.^23^ Briefly, genomic DNA was extracted from the tail of mice. The primer sequences of PCR were directly sourced from the Jackson Laboratory: WT (5′‐TTG CTT TAA ACC CTG CCT GAG CAC‐3′) and mutant miR‐21 (5′‐ACT TCC ATT TGT CAC GTC CTG CAC‐3′). The PCR products were evaluated by agarose gel electrophoresis. A single band obtained at 262 base pair (bp) was identified as WT mice, while a band at 500 bp was identified as miR‐21^−/−^ mice (Figure 1A). In addition, total RNA was also extracted from mouse heart, liver, spleen and palate. miR‐21 expression of miR‐21^−/−^ mice was much lower than that of WT mice (Figure 1A).

### 2.2 Animals and treatments

Six‐week‐old male WT and miR‐21^−/−^ mice (weight: 19 to 21g) were used to establish a model of RME as previously described.^24^ Briefly, an opening loop made from the stainless steel of the orthodontic wire (0.014 inch wire size) (Tomy, Japan) was bonded with a light cured adhesive (3M Unitek, CA) to the first and second maxillary molars on both sides (Figure 1B). It provided an initial force of 0.49 N. Mice without operation served as non‐expansion control.

WT and miR‐21^−/−^ mice were randomly divided into 4 groups, including the control WT group, WT group with expansion force (WT + RME) group, control miR‐21^−/−^ group, and miR‐21^−/−^ group with expansion force (miR‐21^−/−^+RME) group. Mice were euthanized at different time points: 1, 3, 7, 14 or 28 days, with 3 mice in each group at each time point. Animals were weighed at the beginning and the end of the experimental period. The scheme for the animal experiment was shown in Figure S1.

Furthermore, another miR‐21^−/−^ mice with expansion force received agomir‐21, which will increase the expression of miR‐21, at a dose of 80 mg/kg body weight or a comparable volume of PBS (0.2 mL) through intraperitoneal injection every 3 days for 2 consecutive weeks. Measurement of miR‐21 levels was performed after the last injection. Maxillae were removed for further analysis. Agomir‐21 was synthesized by RiboBio Co.

### 2.3 Fluorescence labelling and sample processing

Mice were labelled by fluorescence according to previous methods.^25^ On day 3 and day 13 after the opening loops were applied, alizarin complexone (Sigma, USA) and calcein (Sigma, USA) were injected intraperitoneally at 60 and 20 mg/kg body weight, respectively (Figure S1). Mice were euthanized on the second day after the second dye injection. Maxillae were harvested and fixed with 4% paraformaldehyde. Then, they were embedded in methyl methacrylate without decalcification for slicing hard tissue. Sections of 140 μm were cut with a hard tissue‐slicing machine (Leica, Germany). Double‐labelled sections were viewed by a fluorescence microscope (Leica, Germany).

### 2.4 Histology and histochemistry

Detailed methods were described in the Appendix S1.

### 2.5 Immunohistochemistry

Detailed methods and all antibodies used in this study were described in the Appendix S1.

### 2.6 Cell culture

Detailed methods were described in the Appendix S1.

### 2.7 EdU labelling

Detailed methods were described in the Appendix S1.

### 2.8 3‐(4,5‐dimethylthiazol‐2‐yl)‐2,5‐diphenyltetrazolium bromide (MTT) assay and Cell counting kit‐8 (CCK‐8) assay

Detailed methods were described in the Appendix S1.

### 2.9 Migration assays and Wound Healing Test

Detailed methods were described in the Appendix S1.

### 2.10 Statistical Analysis

The differences between different groups of mice were analysed with Student's *t* test. *P* < .05 was viewed as statistically significant.

## 3 RESULTS

### 3.1 Changes in Body Weight, Palatal Bone Volume and Suture Morphology

During the experiment, the mice that the opening loops had fallen off were not counted in the statistics. The body weights of the mice with activated (expansion) or without opening loops decreased on days 1 and 3 (Figure S2). The loops bonded to maxillary molars may disturb food intake at initial stages, but the mice recovered quickly. At later time points, body weights of these operated mice increased gradually (Figure S2). Therefore, the changes observed following midpalatal suture expansion were unlikely to be caused by systemic physiological responses to the procedure.

Histologically, as Figure 1C and Figure S3 showed, the midpalatal suture of non‐operated groups consisted mainly of cartilage, that is two masses of chondrocytes (arrows showed) covering the edges of palatal bones. During the experimental period, the suture in control animals underwent minor changes related to normal growth with a decrease in the number of chondrocytes (Figure 1D). In the expansion groups, the midpalatal suture was expanded, chondrocytes decreased in numbers (Figure 1D), and the collagen fibres were reoriented across the suture. At the same time, periosteal cells started to migrate into the suture. Periosteal cells in miR‐21^−/−^ mice migrating to the palatal suture need more time than WT mice. As Figure 1C showed, the number of periosteal cells in miR‐21^−/−^ mice is much smaller than WT mice on day 7 after expansion. However, on day 28, the number of migrated cells in miR‐21^−/−^ mice is more than that of WT ones, while WT mice started to reconstruct the normal palatal suture. The suture of miR‐21^−/−^ mice on day 28 (Figure 1C) is similar to that of WT mice on day 14 (Figure S3R). It indicated periosteal cells in miR‐21^−/−^ mice migrating to the palatal suture need more time than WT mice. Bone formation was initially observed at the edges of the palatal bones at day 7 in WT mice. While in miR‐21^−/−^ mice, newly formed bone was observed at day 28, which was later than WT mice. On the oral side, several layers of chondrocytes with a structure similar to the cartilage layers of the original suture can only be detected at day 28 in WT mice.

### 3.2 miR‐21 Deficiency Reduced the Rate of Bone Formation

**Figure 2:**
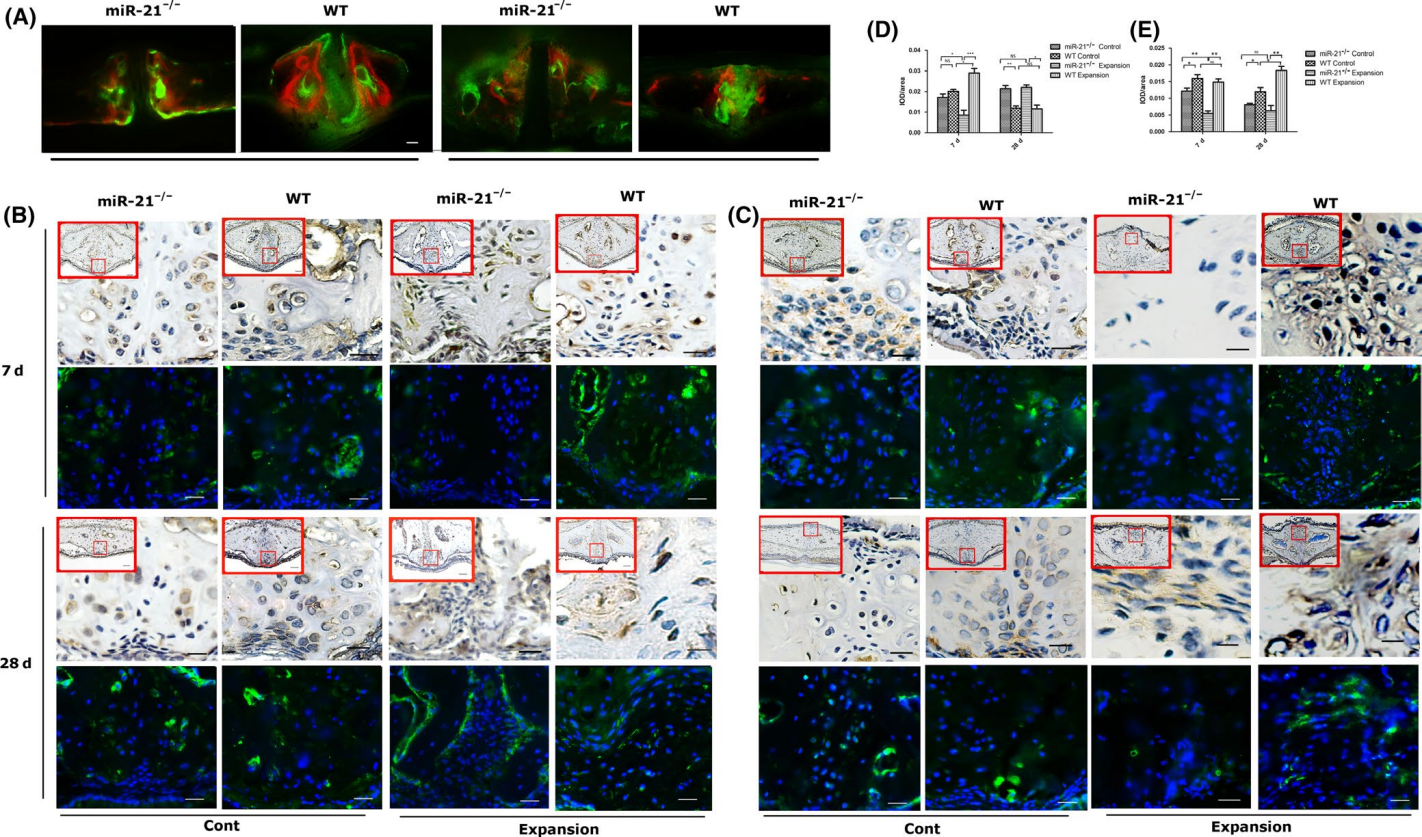
miR‐21 deficiency reduced the rate of bone formation. A, Alizarin complexone and calcein labelling in midpalatal suture after expansion of wild‐type and miR‐21^−/−^ mice. B, D, Alp immunohistochemical staining, immunofluorescence and quantification analysis of midpalatal sutures of control and expansion animals at day 7 and day 28 suggested miR‐21 participated in the bone formation. C, E, Ocn immunohistochemical staining, immunofluorescence and quantification analysis of midpalatal sutures of control and expansion animals at day 7 and day 28. Large red boxed areas show 200 × magnification views. Large pictures showed 400 × magnification views of the small red boxes. Scale bars: 50 μm (200×); 20 μm. (400×). *P < .05; **P < .01; ***P < .001. NS, not significant (*P* > .05). miR‐21, microRNA‐21

To better assess the function of miR‐21 in new bone formation, we labelled bone surfaces with alizarin complexone and calcein during expansion period (Figure S1). As shown in Figure 2A, calcein‐positive bone surface of miR‐21^−/−^ mice was weaker than that of WT mice in control and expansion groups. In order to confirm the finding, we further observed the expressions of alkaline phosphatase (Alp) and osteocalcin (Ocn) using immunohistochemical staining and immunofluorescence.

After force application, the expressions of Alp in both miR‐21^−/−^ and WT mice were reduced during the early days, compared with control groups (Figure S4A). But there were significant elevations of Alp in WT mice after 7 days of expansion and in miR‐21^−/−^ mice after 28 days compared with control groups (Figure 2B,D). Moreover, the expression of Alp was suppressed in miR‐21^−/−^ mice after expansion except 28 days (Figure 2B,D, Figure S4A). Since the expression of Ocn usually occurs in the late osteogenesis process,^26^ a little difference was detected in the expression of Ocn. The expression of Ocn in WT mice after 28 days of expansion was much higher than that of control group (Figure 2C,E). But no significant change was found in miR‐21^−/−^ mice in these groups. The expression of Ocn was significantly suppressed in miR‐21^−/−^ mice (Figure 2C,E, Figure S4B). These findings collectively suggested that miR‐21 deficiency extended the time of bone formation and reduced the rate of bone formation after RME.

### 3.3 miR‐21 Regulated the Bone Resorption and Osteoclastogenesis

**Figure 3:**
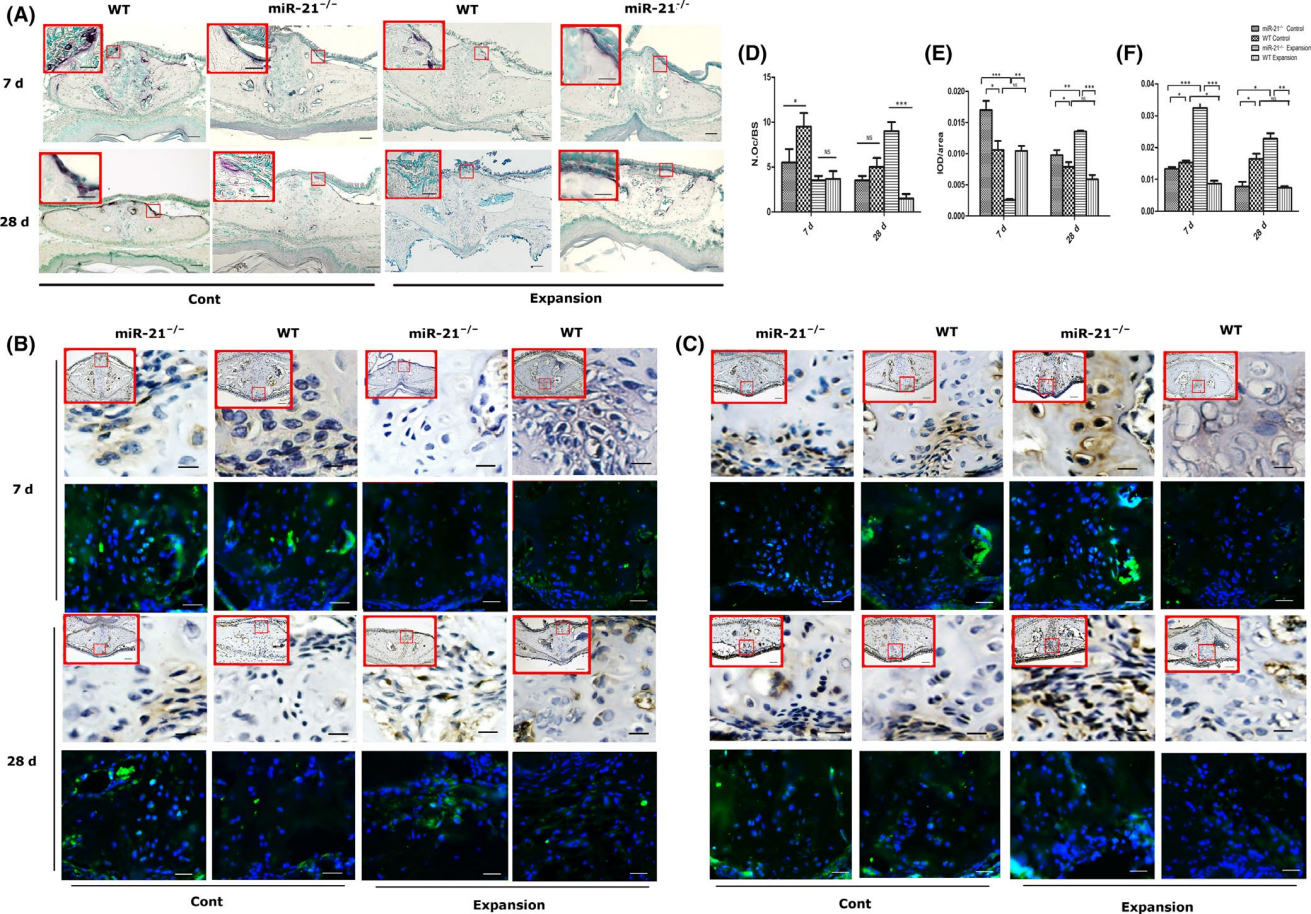
miR‐21 regulated the bone resorption and osteoclastogenesis. (A, D) Tartrate‐resistant acid phosphatase staining in midpalatal suture underwent expansion force in wild‐type and miR‐21^−/−^ mice. Mice were subjected to an expansion force for 7 and 28 d. Red boxed areas indicate osteoclasts analysed on alveolar bone surfaces. Large red boxed areas show 400 × magnification views of the small red boxes. B, E, Opg immunohistochemical staining, immunofluorescence and quantification analysis of midpalatal sutures of control and expansion animals at day 7 and day 28 suggested miR‐21 participates in the bone formation. Large red boxed areas show 200 × magnification views. Large pictures showed 400 × magnification views of the small red boxes. C, F, Rankl immunohistochemical staining, immunofluorescence and quantification analysis of midpalatal sutures of control and expansion animals at day 7 and day 28 suggested miR‐21 participated in the bone formation. Large red boxed areas show 200 × magnification views. Large pictures showed 400 × magnification views of the small red boxes. Scale bars: 50 μm (200×); 20 μm. (400×). *P < .05; **P < .01; ***P < .001. NS, not significant (*P* > .05). miR‐21, microRNA‐21

We examined the potential effects of miR‐21 in bone resorption. Under the physiologic condition, miR‐21 deficiency inhibited bone resorption as shown by tartrate‐resistant acid phosphatase (TRAP) staining (Figure 3A,D, Figure S5A). This result was further supported by increased osteoprotegerin (Opg) expression (Figure 3B,E, Figure S5B,D) and decreased Rankl expression (Figure 3C,F, Figure S5C,E) in miR‐21^−/−^ mice as immunohistochemical staining and immunofluorescence showed. However, during RME with mechanical force, the result was opposite. In miR‐21^−/−^ mice, as TRAP staining showed, there were more osteoclasts in the midpalatal suture areas, mainly on the bone surface. While in WT mice, osteoclasts were less (Figure 3A,D, Figure S5A). According to the immunohistochemical analysis, miR‐21 deficiency downregulated the expression of Opg (Figure 3B,E, Figure S5B,D) and upregulated the expression of Rankl (Figure 3C,F, Figure S5C,E), which was consistent with TRAP staining. The function that miR‐21 regulated bone resorption and osteoclastogenesis was realized by regulating Opg and Rankl.

### 3.4 Agomir‐21 Injection Rescued Decreased Bone Formation and Ratio of Opg/Rankl During Midpalatal Expansion in miR‐21 Deficiency Mice

**Figure 4:**
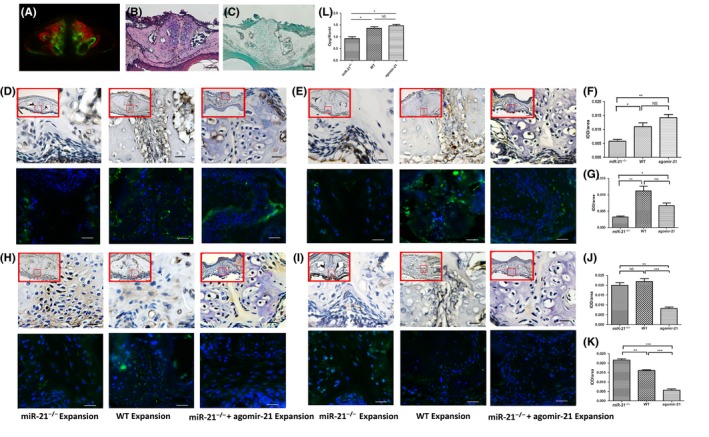
Agomir‐21 injection rescued decreased bone formation and ratio of Opg/Rankl during midpalatal expansion in miR‐21 deficiency mice. Mice were subjected to the expansion force for 14d. A, Alizarin complexone and calcein labelling in midpalatal suture after expansion of agomir‐21‐injected mice. B, HE staining in agomir‐21‐injected mice. C, TRAP staining in midpalatal suture underwent expansion force in agomir‐21‐injected mice. D‐K, Immunohistochemical staining, immunofluorescence and quantification analysis of Alp (D, F), Ocn (E, G), Opg (H, J) and Rankl (I, K). L, The ratio of Opg/Rankl in three different groups. Agomir‐21‐injected mice subjected to midpalatal force for 14 d. Large red boxed areas show 200 × magnification views. Large pictures showed 400 × magnification views of the small red boxes. Scale bars: 50 μm (200×); 20 μm. (400×). *P < .05; **P < .01; ***P < .001. NS, not significant (*P* > .05). miR‐21, microRNA‐21

As mentioned before, miR‐21^−/−^ mice presented decreased bone formation and ratio of Opg/Rankl. To further prove the function of miR‐21, miR‐21^−/−^ mice received agomir‐21 through intraperitoneal injection, which could increase the expression of miR‐21. The result of PCR showed that the expression of miR‐21 in miR‐21^−/−^ mice after injecting agomir‐21 was much higher than that of miR‐21^−/−^ mice (Figure S6). Figure 4B showed periosteal cells in the expanded palatal suture of agomir‐21 injected mice is more than that of miR‐21^‐/‐^ mice (Figure S3M).  Calcein‐positive bone surface of agomir‐21‐injected mice was stronger than that of miR‐21^−/−^ mice after midpalatal expansion (Figure 4A). In order to confirm the finding, the expressions of Alp and Ocn were both detected with immunohistochemical staining and immunofluorescence. After intraperitoneal injection of agomir‐21, the expressions of Alp and Ocn were significantly rescued compared to miR‐21^−/−^ mice (Figure 4D‐G), which suggested that miR‐21 was required for bone formation after midpalatal expansion.

Furthermore, the regulation of osteoclastogenesis was confirmed by both TRAP staining and the ratio of Opg/Rankl (Figure 4L). As Figure 4C presented, fewer osteoclastogenesis formed after injecting agomir‐21. The expressions of Opg and Rankl were lower in mice after injecting agomir‐21 than miR‐21^−/−^ and WT mice (Figure 4H‐K). However, the ratio of Opg/Rankl was higher than that of miR‐21^−/−^ mice, which indicated less bone resorption in agomir‐21‐injected mice. These findings indicated that miR‐21 participated in the process of osteoclastogenesis in midpalatal expansion.

### 3.5 miR‐21 Regulated Proliferation and Migration Ability of BMSCs to Affect the Bone Formation

**Figure 5:**
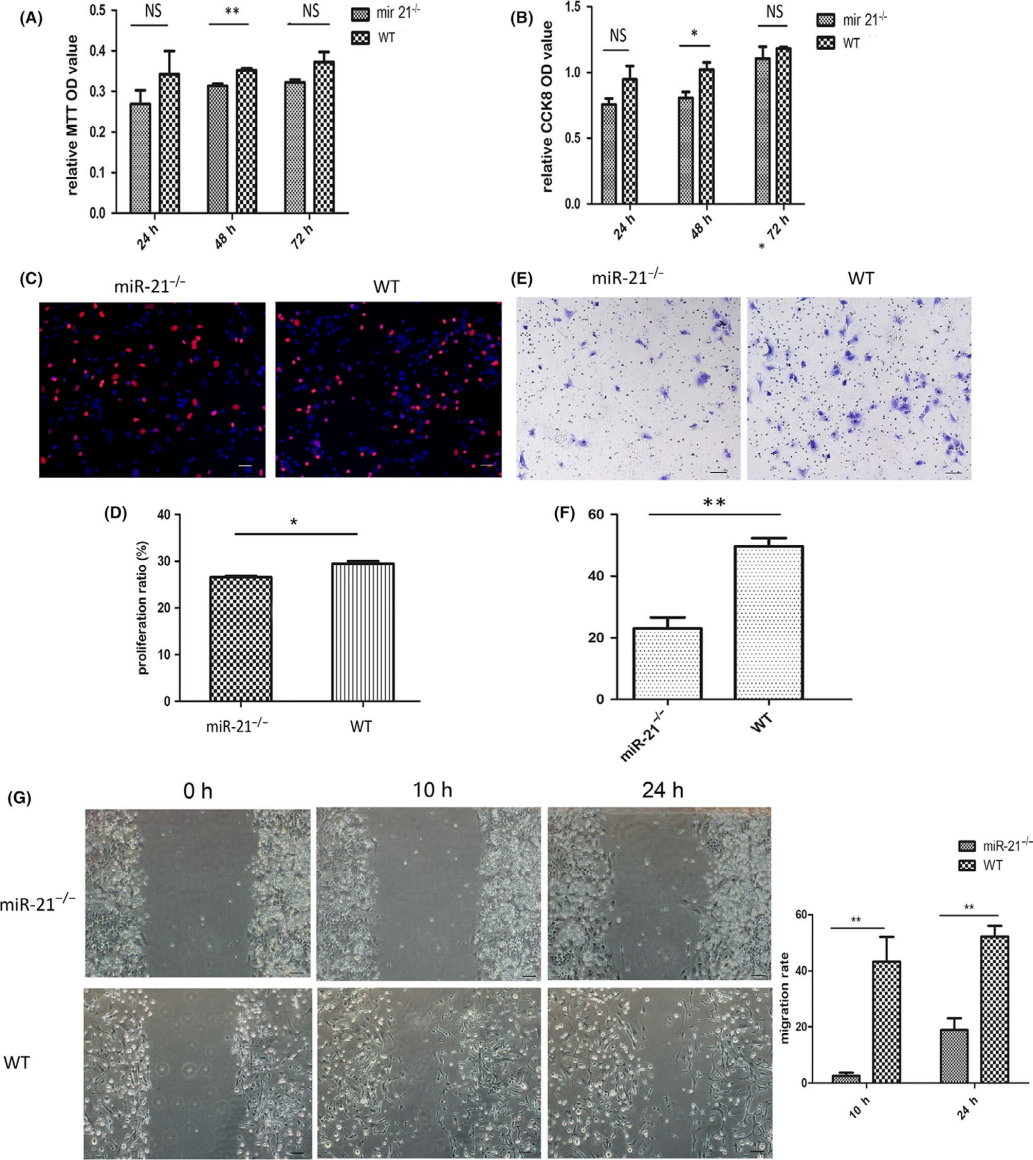
miR‐21 regulated biological characteristics of BMSCs to affect the bone formation. A, B, C, D, Cell proliferation was analysed by MTT (A), CCK8 (B) and EdU (C, D). Bar: 100 μm. E, F, Cell migration was analysed by transwell. Bar: 100 μm. G, Cell migration was analysed by wound healing test. Bar: 135 μm. *P < .05; **P < .01; ***P < .001. NS, not significant (*P* > .05). miR‐21, microRNA‐21

To investigate the mechanism by which miR‐21 regulates bone formation during RME, we cultured the bone mesenchymal stem cells (BMSCs) separated from miR‐21^−/−^ and WT mice (Figure S7 A). The miR‐21 expression of WT mice was much higher than that of miR‐21^−/−^ mice (Figure S7 B). The proliferation of miR‐21‐deficient BMSCs was poorer compared to wild ones through EdU analysis (Figure 5C,D). The finding was further supported by MTT (Figure 5A) and CCK8 (Figure 5B). As Figure 5A‐D showed, in terms of proliferation, there was a significant difference between these two kinds of BMSCs in 48 h. Moreover, we examined the migration ability of two kinds of BMSCs by transwell migration assays and wound healing test (Figure 5E‐G). miR‐21 deficiency led to significantly decreased migrated cells (Figure 5E,F). In wound healing test, the distance of cell migration in wild cells was longer than miR‐21‐deficient ones, which can be obviously detected in 24 h (Figure 5G). These findings suggested that miR‐21 regulated proliferation and migration ability of BMSCs to regulate bone formation during RME.

## 4 DISCUSSION

Our previous study showed that miR‐21 was related with mechanical force–induced osteogenesis.^19^ Despite in vitro result showed miR‐21 is an osteogenesis promoter in bone marrow mesenchymal stem cells and PDLSCs,^19^ the in vivo function of miR‐21 in maxillary expansion has not been elucidated. Here, the function of miR‐21 in bone formation and resorption was investigated after maxillary expansion in mice through micro‐CT, HE staining, TRAP staining, immunohistochemistry and fluorescence labelling.

Maxillary expansion, a method with mechanical force, has been widely used in orthodontics therapy, which was previously described as histologic changes during the process. Numerous studies have reported that in the early stage of bone reorganization after expansion, many osteoblasts and fibroblasts exist in the suture, the conjunction tissue is loose, blood vessels are large and disorganized, and irregular trabeculae is bordered with large medullary spaces; in the stage of maturation, fewer osteoblasts and fibroblasts are observed in the suture, the conjunction tissue is dense with fibers arranged in bundles, blood vessels are small and evenly distributed, and regular trabeculae is bordered with small medullary spaces.^13^, ^27^, ^28^

The expanded palatal suture in this experiment presented similar changes. miR‐21^−/−^ expansion group presented more loose conjunction tissue than WT expansion group in the early stage of expanding. After 14 days, the conjunction tissue of miR‐21^−/−^ expansion group started to become dense, with fibres arranged more perpendicularly. In WT expansion group, a dense conjunction tissue was observed in the suture area. The osteoblasts and fibroblasts of WT expansion group were much more than those of miR‐21^−/−^ expansion group. After 28 days, in miR‐21^−/−^ expansion group, the conjunction tissue was dense with fibres in various directions, with more osteoblasts and fibroblasts in this area. The conjunction tissues in WT expansion group were more similar to the configuration of the unexpanded suture, with fewer osteoblasts and fibroblasts compared to that in 14 days, which suggested a more advanced stage of maturation. Therefore, knocking out miR‐21 can prolong the time of bone regeneration. Chen and his colleagues^22^ reported that miR‐21 contributes to orthodontic force–induced osteoblastogenesis and alveolar bone formation during orthodontic tooth movement, which is consistent with our result in terms of bone formation which were induced by strength.

Moreover, we confirmed that miR‐21 deficiency blocked the bone remodelling under physiologic conditions, not only bone resorption but also bone formation. Previous studies showed that miR‐21 could promote osteogenesis and osteoclast differentiation.^29^, ^30^ Huiskes et al^31^ have reported that bone resorption cells (osteoclasts) and bone formation cells (osteoblasts) normally balance bone mass in a coupled homeostatic process of remodelling. However, when the external mechanical force breaks the autologous balance between osteogenesis and osteoclastogenesis, the RANKL/OPG ratio and osteoclasts increased. Previous studies have reported that with the action of mechanical force, many factors such as smad5 can affect the osteoclast differentiation.^32^ Li et al 33 reported miR‐21 could increase the expression of smad5, which will downregulate the expression of osteoclast serum markers.^32^ Therefore, with mechanical force, smad5 may be one of the factors regulated by miR‐21 to affect bone resorption. In our study, bone resorption mainly started from bone surface where osteoclasts were mainly distributed.

We further observed that the number of periosteal cells, which can promote osteogenic differentiation,^24^, ^34^ in WT mice was much more than that of miR‐21^−/−^ mice, suggesting that miR‐21 deficiency might affect the biological characteristics of cells. In vitro, it revealed that proliferation and migration ability of miR‐21‐deficient cells were poorer, which was consistent with previous studies.^35^, ^36^ Since decreased proliferation and migration ability of periosteal cells lead to decreased osteogenesis, less osteoblasts and fibroblasts in miR‐21^−/−^ expansion group can be exactly explained in this study. In addition, many researches have proved that miR‐21 in BMSCs is benefit for osteogenesis both in vitro and in vivo.^37^, ^38^ For example, miR‐21 overexpressing can promote osteogenic differentiation and accelerate fracture healing. Our study is consistent with previous studies.

In summary, our findings showed that miR‐21 is related with the changes in biological characteristics of cells and the maturation of newly formed bone in the process of RME.

## CONFLICT OF INTEREST

All authors declare that they have no potential conflicts of interest.

## AUTHOR CONTRIBUTIONS

M. Li contributed to design, data acquisition, analysis and interpretation, and drafted and critically revised the manuscript; Z. Zhang and X. Gu contributed to data acquisition and analysis, and critically revised the manuscript; Y. Jin, C. Feng and S. Yang contributed to data analysis, and critically revised the manuscript; F. Wei contributed to conception, and critically revised the manuscript. All authors gave final approval and agreed to be accountable for all aspects of the work.

## Data Availability

The data used to support the findings of this study are available from the corresponding author upon request.
