# Supplementary material for: MicroRNA‐21 affects mechanical force–induced midpalatal suture remodelling
Source: Cell Prolif. 2019 Nov 12;53(1):e12697. doi: 10.1111/cpr.12697 (PMC6985676; doi:10.1111/cpr.12697)
Supplement: Supplementary file 1 [file CPR-53-e12697-s001.docx]

**MicroRNA-21 affects mechanical force–induced midpalatal suture remodelling**

**Running head： Effect of miR-21 on midpalatal suture remodelling**

**Authors:**

Mengying Li^1,2^, Zijie Zhang^1,2^, Xiuge Gu^1,2^, Ye Jin^1,2^, Cheng Feng^3^, Shuangyan Yang^1,2^, FulanWei^1,2*^

**Affiliations**:

1. Shandong Provincial Key Laboratory of Oral Tissue Regeneration, School of Stomatology, Shandong University, Jinan, People's Republic of China.

2. Department of Orthodontics, School of Stomatology, Shandong University, Jinan, People's Republic of China.

3. Jinan Hospital of Traditional Chinese Medicine, Jinan, People's Republic of China.

***Corresponding author:**

Fulan Wei; Department of Orthodontics, Shandong Provincial Key Laboratory of Oral Tissue Regeneration, School of Stomatology, Shandong University, Wenhua Xi Road No. 44-1, Jinan, Shandong 250012, People's Republic of China; E-mail: weifl@sdu.edu.cn.

**Appendix:** Appendix Materials & Methods, Appendix References, 7 Appendix Figures are available.

**APPENDIX MATERIALS & METHODS**

**Histology and histochemistry**

Maxillaes were decalcified using 10% ethylene diamine tetraacetic acid (pH, 7.2 to 7.4) and embedded in paraffin. Paraffin-embedded specimens were then sectioned into 5μm. For morphological observation, sections were stained with haematoxylin and eosin (HE). To quantify osteoclasts, tartrate-resistant acid phosphatase (TRAP) staining was performed on sections from each group. Multinucleated TRAP-positive cells attached to alveolar bone surfaces were counted under high magnification. The number of osteoclasts from each group was subjected to statistical analysis.

**Immunohistochemistry**

The expression of osteogenesis-related genes, such as Alp and Ocn, and bone resorption–related genes, such as Rankl and Opg, were examined by immunohistochemistry, respectively. Briefly, 5μm sections from each sample were deparaffinized, treated by 0.25% trypsin (Solarbio, China) for 30 min at 37 °C for antigen retrieval, washed and treated by 3% hydrogen peroxide for 20 min at 37 °C. Sections were blocked with 5% bovine serum albumin (Sigma-Aldrich) in phosphate-buffered saline for 2 hours at room temperature.

Sections were then stained with rabbit anti-mouse Alp, Ocn, Rankl, Opg　primary antibodies (Abcam, UK) overnight at 4°C, each at a concentration of 1:200, followed by a horseradish peroxidase–conjugated goat anti-rabbit secondary antibody for 30 min at room temperature at a concentration of 1:200. Subsequently, a horseradish peroxidase–based diaminobenzidine was used to detect immunoactivity, followed by counterstaining with haematoxylin (Solarbio, China). Quantification of positive-stained cells over the whole periodontal area was based on at least 3 fields per animal, via Image-Pro Plus 6.0 software.

**Cell culture**

Bone marrow was flushed out of the femur and tibia cancellous bone as previously described (Yuan et al. 2014). Then, they were dispersed with Dulbecco’s modified Eagle’s medium (DMEM; Hyclone, USA) supplemented with 15% foetal bovine serum (FBS; Gibco, USA), 100 U/ml penicillin and 100 μg/ml streptomycin (Invitrogen, USA). Primary cells were seeded in a 75 cm^2^ culture flask at 37 °C in 5% carbon dioxide. The cells were digested with 0.25% trypsin (Hyclone, USA) when cell clones reached over 80% confluence. Then, cells were cultured in DMEM supplemented with 10% FBS, 100 U/ml penicillin and 100 μg/ml streptomycin. The medium was changed every 2-3 days. Cells were passaged two to three times in the same medium before being used for the following experiments.

**EdU labelling**

Cells at 2nd passage were used in the present study. BMSCs were seeded into a 24-well plate at 6×10^4^ cells/well in DMEM with 10% FBS and incubated at 37°C. Twenty-four hours later, 200μl of EdU reagent (RiboBio, China) was added to the medium at 50 μM. After 2 hours, cells were fixed with paraformaldehyde, washed twice with phosphate-buffered saline (PBS), and incubated in 0.5% Triton X-100 in PBS for 10 min. For EdU staining, cells were incubated with Apollo reagent for 30 min at room temperature in the dark. 0.5% Triton X-100 in PBS was then used to wipe off the Apollo reagent. For DNA staining, cells were further stained with Hoechst 333424 for 30 min at room temperature in the dark.

The stained cells were examined and photographed with fluorescence microscope. To determinate the percentage of EdU-positive cells, the number of red-fluorescent (Apollo-stained) cells was divided by the number of blue-fluorescent (Hoechst 333424-stained) cells. The experiment was performed in triplicate, and data presented in results were the average of three independent experiments.

**3-(4,5-dimethylthiazol-2-yl)-2,5-diphenyltetrazolium bromide (MTT)assay**

BMSCs were seeded into 96-well plates at a cell density of 5000 cells/well. Then, the cells were cultured in DMEM with 10% FBS at 37 °C for 24 h, 48 h and 72 h, respectively. Subsequently, the culture medium was replaced with 5 mg/mL MTT solution (Sigma-Aldrich, USA) diluted in PBS. The plates were incubated again for 4 h at 37 °C, and a volume of 150 μL DMSO was added to each well. Optical density (OD) was measured at 490 nm (*A_490_*).

**Cell counting kit-8 (CCK-8) assay**

Growth of the BMSCs was evaluated using a CCK-8 (Beyotime Institute of Biotechnology, China) assay according to the manufacturer’s protocol. Cells were seeded into 96-well plates at a density of 5000 cells/well and cultured for 24 h. Then, 10 μl of CCK-8 solution was added into the wells and cells were incubated at 37 °C for 1.5 h. Subsequently, OD of cells was determined at 450 nm (*A_450_*) using a microplate reader (Bio-Rad, USA).

**Migration assays**

Cell capability of migration was examined by 24-well transwell chamber following the instructions of the manufacturer. To evaluate cell migration, cells were plated into top chamber of transwell migration chamber at a density of 6×10^4^ cells/well in 100 μl serum-free media and 500 μl medium containing 10% FBS was added into the lower chamber. Following incubation at 37 °C for 24 h, the filter was gently removed from the chamber and the remaining cells on the upper surface of the filter were wiped off with a cotton swab. The cells that had migrated to the lower surface of the filter were fixed with 4% paraformaldehyde for 10 min and stained with 0.5% crystal violet for 15 min. The number of migrated cells was counted from five random fields (× 200) under a microscope (Olympus, Japan).

**Wound Healing Test**

BMSCs were seeded into 6-well plates at a density of 4×10^5^ cells/well in complete medium and grew to a confluence of 80%. Subsequently, a scratch of cells was inflicted using a sterile 1000 µl pipette tip. Gentle washing was carried out twice using PBS to remove debris. The cells were incubated with 1ml of serum-free medium for 24h. Images of wound coverage were observed at ×80 magnification (Olympus, Japan) at 0h, 10h and 24h

**APPENDIX REFERENCES**

Yuan W, Liu W, Li J, Li X, Sun X, Xu F, Man X, Fu Q. 2014. Effects of BMSCs interactions with adventitial fibroblasts in transdifferentiation and ultrastructure processes. Int J Clin Exp Pathol. 7(7): 3957-3965.

**APPENDIX FIGURES & APPENDIX FIGURE LEGENDS**


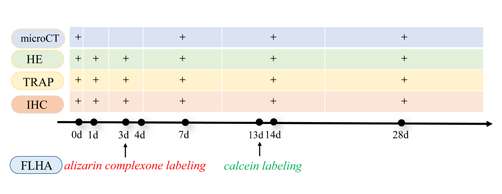


**Appendix Fig. 1** Time schedule of the present study. HE, HE staining; TRAP, tartrate-resistant acid phosphatase staining; IHC, immunohistochemistry; FLHA, fluorochrome labelling histomorphometrical analysis.


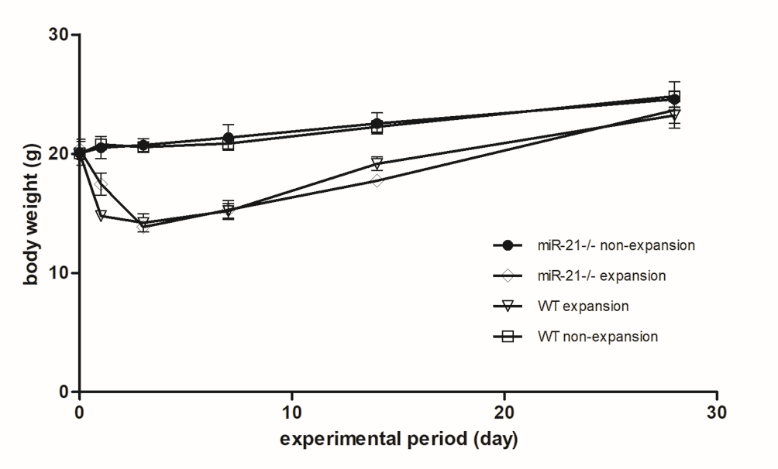


**Appendix Fig. 2** Changes in body weight during experimental period**.** Body weight curves of control and experimental groups. The body weights of expansion were statistically lower than the control groups at day 1, day 3 and day 14. However, the body weights recovered after 14d.

**
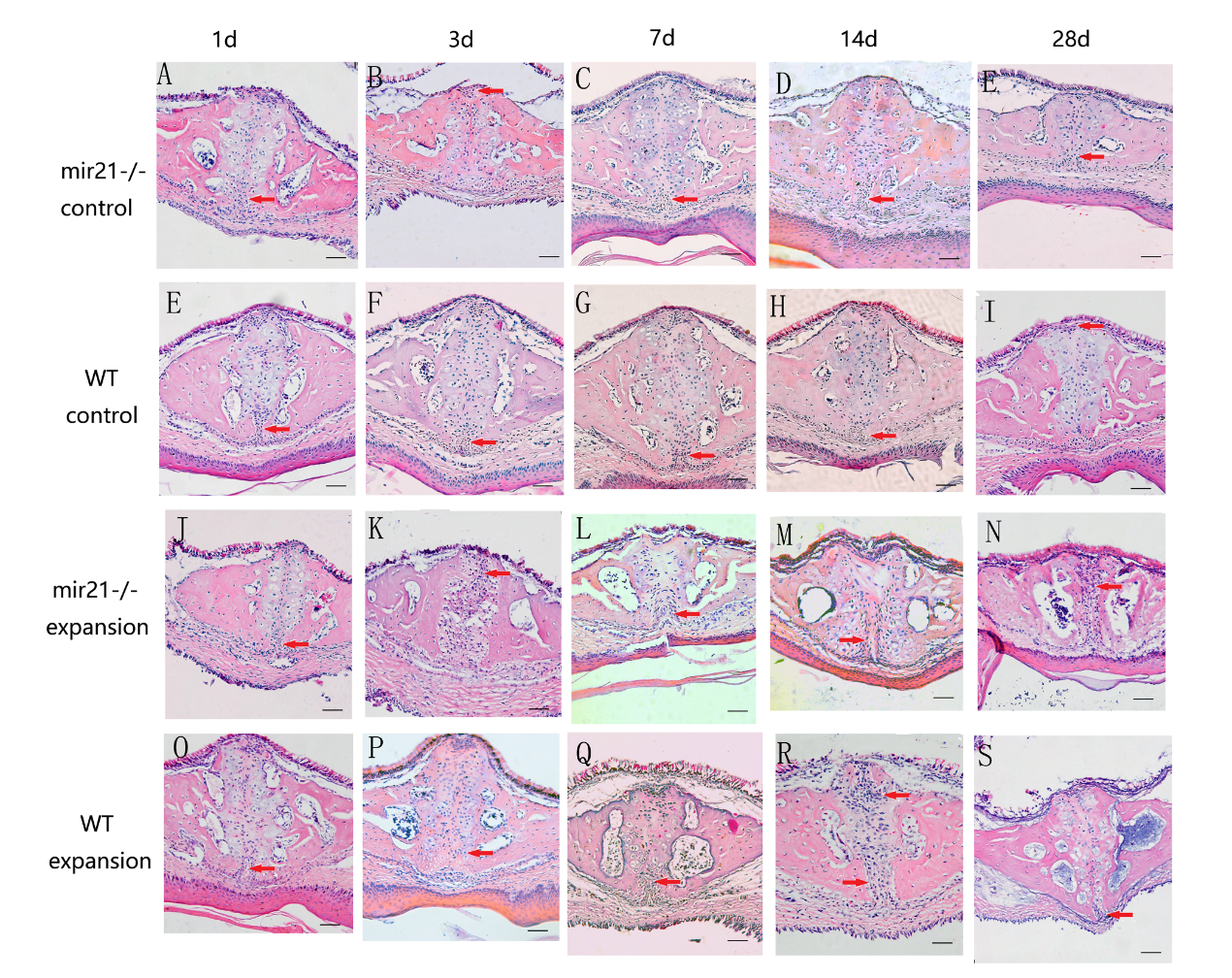
**

**Appendix Fig. 3** Haematoxylin and eosin staining of every group in control and expansion animals at days 1, 3, 7, 14 and 28. Red arrows point to the periosteum within the oral region of the midpalatal suture. Scale bar: 50μm.


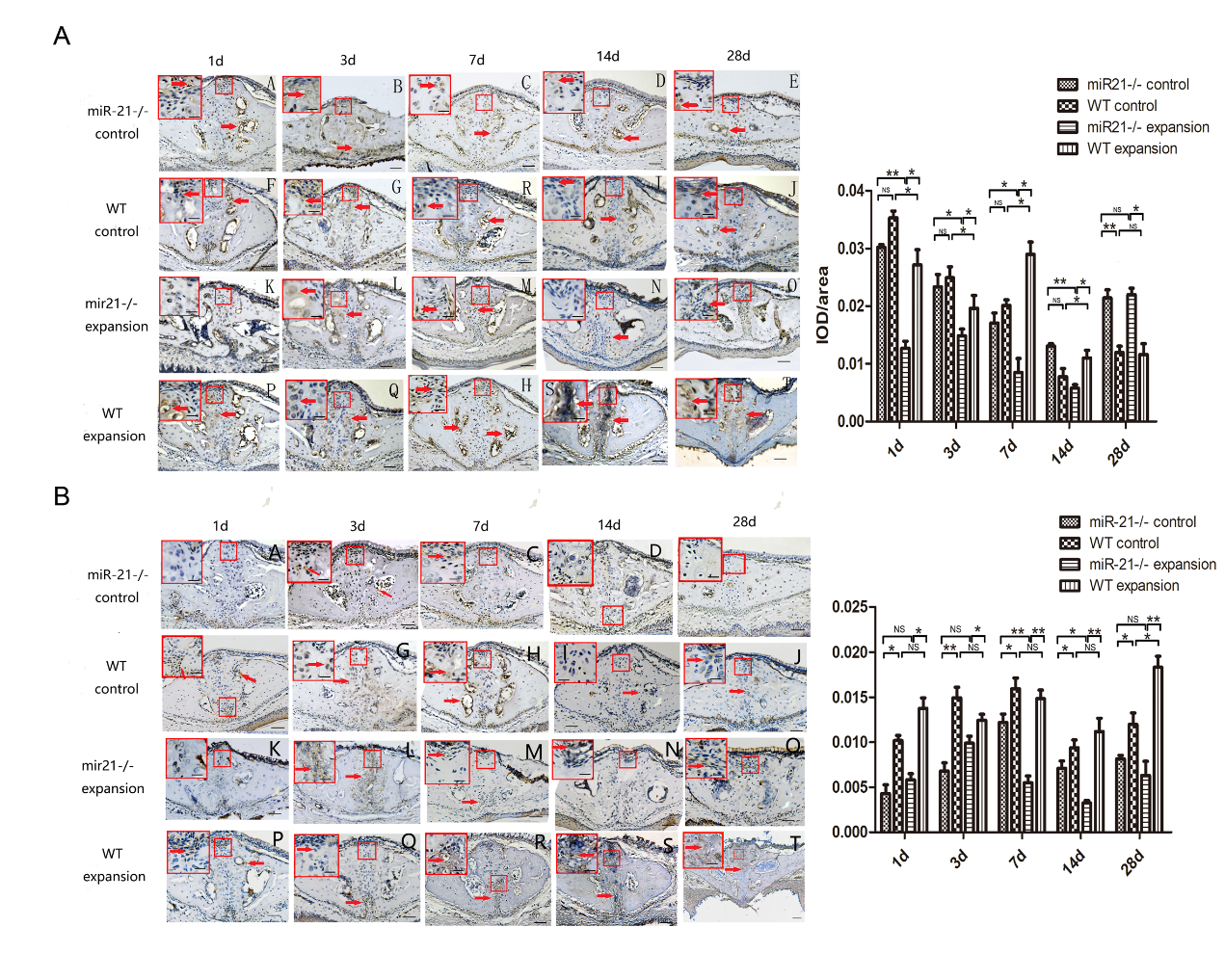


**Appendix Fig. 4** Immunohistochemical staining and quantification analysis of alkaline phosphatase (Alp) **(A)** and osteocalcin (Ocn) **(B)**. Large red boxed areas show higher-magnification views of the small red boxes. Red arrows point to the Alp/Ocn-positive cells. Bars: 50 μm. **P*< 0.05; ***P*< 0.01; ****P*< 0.001. NS, not significant (*P*> 0.05). miR-21, microRNA-21.


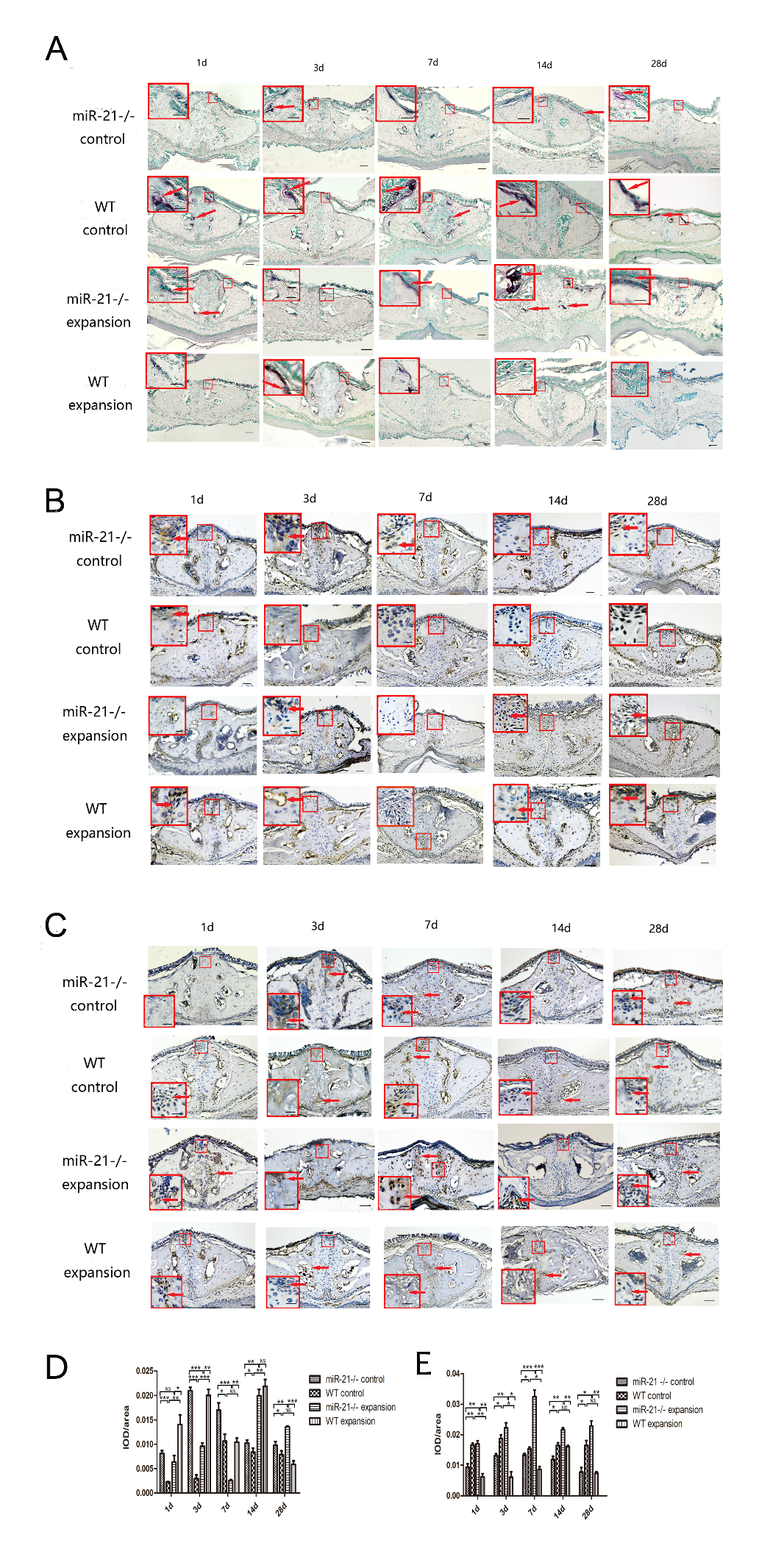


**Appendix Fig. 5** miR-21 regulated the bone resorption and osteoclastogenesis. **(A)** Tartrate-resistant acid phosphatase (TRAP) staining in midpalatal suture underwent expansion force in wild-type (WT) and miR-21^-/-^ mice. Large red boxed areas show higher-magnification views of the small red boxes. Red arrows point to the osteoclast cells. **(B, C, D, E)** Immunohistochemical staining and quantification analysis of osteoprotegerin (Opg) **(B, D)**, and receptor activator of nuclear factor-κ B ligand (Rankl) **(C, E)**. Large red boxed areas show higher-magnification views of the small red boxes. Red arrows point to the Opg/Rankl-positive cells. Bars: 50 μm. **P*< 0.05; ***P*< 0.01; ****P*< 0.001. NS, not significant (*P*> 0.05). miR-21, microRNA-21.


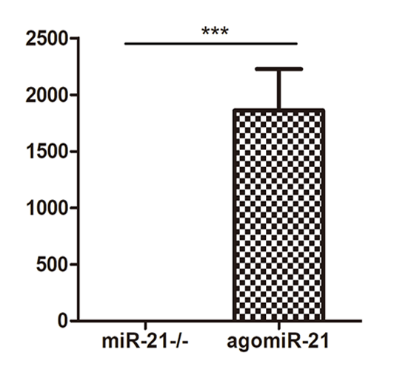


**Appendix Fig. 6** qRT-PCR analysis of the expression levels of miR-21 in the palate tissue of miR-21^-/-^ mice and agomir-21-injected mice. ****P*< 0.001.


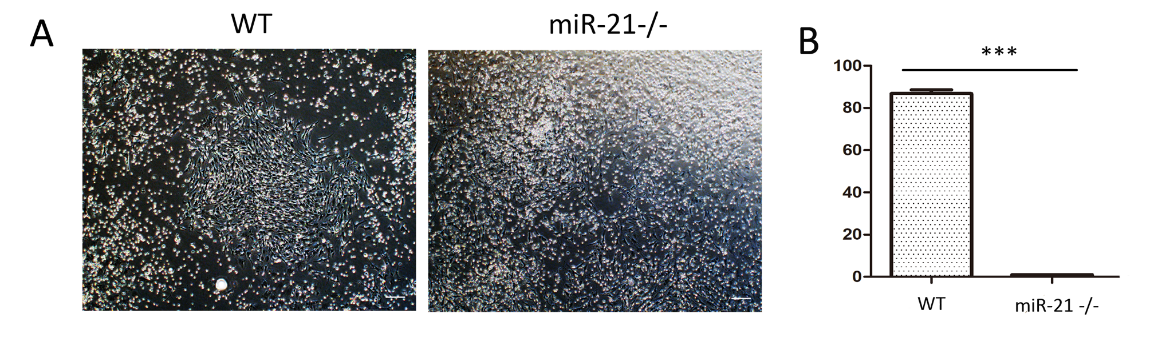


**Appendix Fig. 7 (A)** Images of BMSCs derived from WT and miR-21^-/-^ mice cultured in normal media photographed by optical microscopy. **(B)** qRT-PCR analysis of miR-21 expression in two types of cells. ****P*<0.001. Scale bar: 200μm.
